# TP53-Induced Glycolysis and Apoptosis Regulator (TIGAR) Is Upregulated in Lymphocytes Stimulated with Concanavalin A

**DOI:** 10.3390/ijms22147436

**Published:** 2021-07-11

**Authors:** Helga Simon-Molas, Xavier Vallvé-Martínez, Irene Caldera-Quevedo, Pere Fontova, Claudia Arnedo-Pac, Anna Vidal-Alabró, Esther Castaño, Àurea Navarro-Sabaté, Núria Lloberas, Ramon Bartrons, Anna Manzano

**Affiliations:** 1 Departament de Ciències Fisiològiques, Facultat de Medicina i Ciències de la Salut, Universitat de Barcelona, C/Feixa Llarga, s/n, L’Hospitalet de Llobregat, 08907 Barcelona, Spain; hsimon@ub.edu (H.S.-M.); xvallvma7@alumnes.ub.edu (X.V.-M.); annavidal@ub.edu (A.V.-A.); rbartrons@ub.edu (R.B.); 2 Molecular Imaging Department, Beatson Institute—Cancer Research UK, Glasgow G61 1BD, UK; irecal@hotmail.es; 3 Departament de Nefrologia, Hospital Universitari de Bellvitge, IDIBELL, 08907 Barcelona, Spain; pfontopa7@gmail.com (P.F.); nlloberas@ub.edu (N.L.); 4 Institute for Research in Biomedicine (IRB Barcelona), The Barcelona Institute of Science and Technology (BIST), 08036 Barcelona, Spain; claudia.arnedo@irbbarcelona.org; 5 Centres Científics i Tecnològics, Universitat de Barcelona, 08907 Barcelona, Spain; mcastano@ub.edu; 6 Departament d’Infermeria Fonamental i Medicoquirúrgica, Facultat de Medicina i Ciències de la Salut, Universitat de Barcelona, 08907 Barcelona, Spain; aureanavarro@ub.edu

**Keywords:** TIGAR, lymphocytes, glycolysis, PPP, ROS, PI3K/AKT, autophagy

## Abstract

The glycolytic modulator TP53-Inducible Glycolysis and Apoptosis Regulator (TIGAR) is overexpressed in several types of cancer and has a role in metabolic rewiring during tumor development. However, little is known about the role of this enzyme in proliferative tissues under physiological conditions. In the current work, we analysed the role of TIGAR in primary human lymphocytes stimulated with the mitotic agent Concanavalin A (ConA). We found that *TIGAR* expression was induced in stimulated lymphocytes through the PI3K/AKT pathway, since Akti-1/2 and LY294002 inhibitors prevented the upregulation of *TIGAR* in response to ConA. In addition, suppression of *TIGAR* expression by siRNA decreased the levels of the proliferative marker PCNA and increased cellular ROS levels. In this model, TIGAR was found to support the activity of glucose 6-phosphate dehydrogenase (G6PDH), the first enzyme of the pentose phosphate pathway (PPP), since the inhibition of *TIGAR* reduced G6PDH activity and increased autophagy. In conclusion, we demonstrate here that TIGAR is upregulated in stimulated human lymphocytes through the PI3K/AKT signaling pathway, which contributes to the redirection of the carbon flux to the PPP.

## 1. Introduction

T lymphocytes, also known as T cells, are key players in the adaptative immune system. They participate in cellular immunity by targeting infected or cancerous cells, but also in humoral immunity by recruiting and activating other immune cells such as macrophages or B lymphocytes [1,2]. T lymphocytes are produced in the bone marrow and continue their development in the thymus, where they mature into naïve T cells. Once they enter circulation, naïve T cells are maintained in a resting or quiescent state by T cell receptor (TCR) stimulation through self-peptides presented by major histocompatibility complex (MHC) molecules, as well as interleukin 7 (IL-7) signalling [3,4,5]. Naïve T cells harbor the capacity for antigen recognition, but still lack effector capabilities. Upon encountering an external antigen, which generally occurs in lymphoid tissues, naïve T cells differentiate into effector T cells and long-lived memory T cells. Activation of T lymphocytes is mediated by a process known as quiescence exit, which drives their clonal expansion and development of effector functions. During this phase, T cell metabolism is quickly reprogrammed to accomplish the increased energy and biosynthetic precursor demand required for their proliferation and growth. This metabolic reprogramming is based on an increment of the glycolytic flux, which results in higher ATP production, together with increased mitochondrial metabolism, glutaminolysis and lipid synthesis. Besides, the high glycolytic rate allows some of the glycolytic intermediates to enter the pentose phosphate pathway (PPP) to promote nucleotide biosynthesis [5]. After clonal expansion, metabolically reprogrammed T lymphocytes leave the lymphoid tissues to the peripheral tissues where the recognized antigen is located.

The first metabolic changes occurring during lymphocyte activation include enhanced expression of glucose transporter 1 (*GLUT1*) and key glycolytic enzymes such as hexokinase-II (*HK-II*) [6]. In parallel, these changes are followed by a fast increase in the intracellular concentration of fructose 2,6- bisphosphate (Fru-2,6-P_2_), the most potent allosteric activator of the glycolytic enzyme phosphofructokinase-1 (PFK-1) [7,8,9]. The synthesis and degradation of Fru-2,6-P_2_ is catalyzed by 6-phosphofructo-2-kinase/fructose-2,6-bisphosphatase (PFK-2/FBPase-2), which can be encoded by four different genes, giving rise to four different isoenzymes [8]. The isoenzyme with the highest kinase/bisphosphatase ratio, and therefore the one contributing to a largest increase in the glycolytic flux, is PFKFB3. Previous work from our group and others have shown the contribution of PFKFB3 to the activation of human and mouse T cells [10,11]. We showed that *PFKFB3* mRNA and protein levels were increased upon treatment of human T-lymphocytes with mitogenic stimuli that induced activation and proliferation of these cells, concomitantly with *GLUT-1* and *HK-II* expression.

The TP53-induced glycolysis and apoptosis regulator (*TIGAR*), also known as *c12orf5*, is a p53 regulated gene that is activated by low levels of stress and is overexpressed by malignant cells from different origins [8,12,13]. TIGAR is mainly located in the cytoplasm and belongs to the histidine phosphatase superfamily of proteins. Its bisphosphatase catalytic domain hydrolyses Fru-2,6-P_2_ into fructose-6-phosphate (Fru-6-P) and Pi [12,14] and shares high similarity with the bisphosphatase domain of PFK-2/FBPase-2. Therefore, TIGAR activity has also an impact on Fru-2,6-P_2_ levels and glycolytic flux, reversing PFKFB3 activity and decreasing glycolysis at the expense of the pentose phosphate pathway (PPP) [8,12,15,16]. Additionally, Fru-6-P can be converted to glucose-6-phosphate (Glu-6-P), the substrate of the first enzyme in the PPP, and glucose-6-phosphate dehydrogenase (G6PDH) [17]. PPP synthesizes ribose-5-phosphate (Ribose-5-P), the central molecule for DNA replication, and NADPH, which is essential for the regeneration of oxidized to reduced glutathione. Increasing antioxidant potential renders cells more resistant to ROS-induced apoptosis. By decreasing ROS, TIGAR has been described as a tumor suppressor in the intestinal epithelium [18]. The inhibition of TIGAR has been described to sensitize malignant cells to H_2_O_2_- and radiotherapy-induced cell death [19,20,21,22,23].

Studies performed in tumoral cells show that the expression levels of *PFKFB3* and *TIGAR* can be increased at the same time [24]. Promoting glycolysis and the PPP can confer an advantage to proliferative cells with high glucose avidity in a context of high demand of ATP and nucleotides. The aim of the present work was to investigate the role of TIGAR in stimulated human lymphocytes, focusing on carbon metabolism and ROS homeostasis.

## 2. Results

### 2.1. TIGAR Is Induced in ConA-Activated Human Lymphocytes

In a previous study, our group showed that the mitogenic agent Concanavalin A (ConA) induced T cell activation and proliferation in parallel to enhanced expression of *PFKFB3* and increased Fru-2,6-P_2_ concentration levels, in a PI3K/Akt-dependent manner [10]. The data obtained proved the increased presence of the early activation marker CD25, also known as interleukin-2 receptor alpha chain (IL2RA), in the membrane of ConA-treated lymphocytes. Besides, untreated cells showed higher carboxyfluorescein succinimidyl ester (CFSE) fluorescence levels compared to ConA-treated T-lymphocytes, indicating increased proliferation of T cells in the presence of ConA. The effect on proliferation was confirmed by increased PCNA protein levels in ConA-treated cells [10].

In the present study, a similar experimental setup based on ConA treatment was used to further study the metabolic changes taking place in stimulated lymphocytes, with a focus on TIGAR.

**Figure 1:**
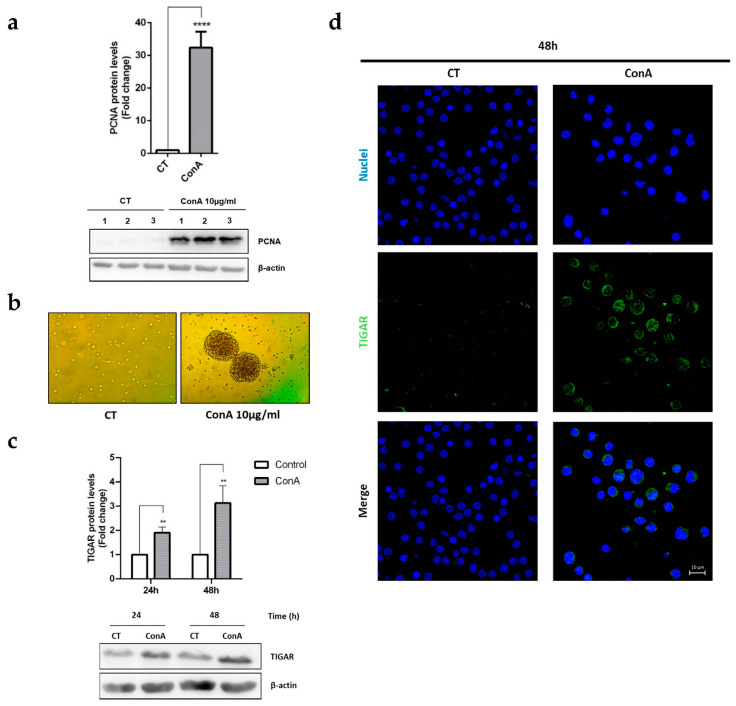
TIGAR is induced in parallel to the proliferation marker PCNA in ConA-stimulated human lymphocytes. (**a**) Western blot image and quantification of PCNA levels normalized to β-actin in response to 10 µg/mL ConA at 48 h. Representative images of three different samples are shown. (**b**) Optical microscopy images of control and ConA-treated T lymphocytes at 48 h. (**c**) Western blot analysis of TIGAR normalized to β-actin in 10 µg/mL in ConA-treated and untreated (Ct) lymphocytes at 24 h and 48 h. A representative image is shown. (**d**) Image of a representative immunofluorescence analysis of TIGAR in ConA-treated lymphocytes at 48 h. Nuclei were detected with DRAQ5 (Thermo Fisher). All data are presented as a mean fold change relative to untreated cells ± SEM. Differences were calculated with two-tailed unpaired Student’s *t*-test with a normal-based 95% CI. Significant *p* values are indicated; ** *p* < 0.01; **** *p* < 0.0001; *n* = 3.

First, PCNA protein levels, which are indicative of lymphocyte cell proliferation, were analysed by Western blot. PCNA was significantly increased in ConA-treated lymphocytes after 48 h of treatment (Figure 1a). Similarly, T lymphocyte clustering after activation was confirmed by optical microscopy images (Figure 1b).

The effects of ConA on TIGAR protein levels were analysed by Western blot. TIGAR was significantly upregulated 24 h and 48 h after ConA treatment in exposed compared to untreated cells. The maximum levels of expression of TIGAR were obtained after 48 h of ConA treatment (Figure 1c).

The subcellular localization of TIGAR was assessed by immunofluorescence, which confirmed the presence of higher levels of the protein after 48 h of ConA treatment. We also found that TIGAR was mainly located in the cytoplasm of unstimulated lymphocytes, while its expression was increased in both the cytoplasm and the nucleus of activated lymphocytes (Figure 1d).

### 2.2. TIGAR Induction by ConA Is Mediated by PI3K/Akt Signaling Pathway

**Figure 2:**
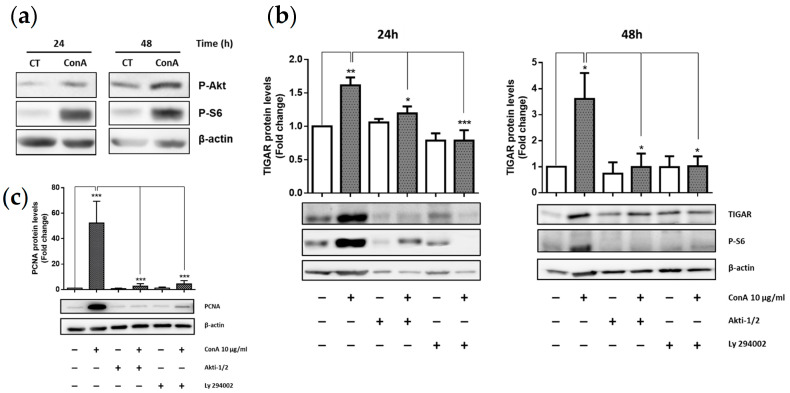
PI3K/Akt inhibition prevents TIGAR and PCNA induction in ConA-stimulated lymphocytes. (**a**) Western blot analysis of P-Akt and P-S6 incubated with 10 µg/mL ConA normalized to β-actin. (**b**) Western blot analysis and quantification of TIGAR and P-S6 incubated with PI3K/Akt inhibitors (Akti-1/2 and Ly 294002) at a concentration of 10 µM and 20 µM respectively and normalized to β-actin. (**c**) PCNA protein levels in response to the aforementioned conditions at 48 h. All data are presented as a mean fold change relative to untreated cells ± SEM. Differences were calculated with two-way ANOVA and Tukey’s multiple comparisons test with a normal-based 95% CI. Significant *p* values are indicated; * *p* < 0.05; ** *p* < 0.01; *** *p* < 0.001, *n* = 3 treated with ConA.

The PI3K/Akt pathway has been described as a driver of important metabolic changes, including PFKFB3 upregulation in T lymphocytes during their activation and proliferation [24,25]. Therefore, it was of interest to analyse whether *TIGAR* expression was also under the control of this signaling pathway in ConA-treated cells. First, we checked PI3K/Akt activation 24 h and 48 h after ConA treatment. Significant increased protein levels of P-Akt (S473) and P-S6 were detected in ConA-treated cells, whereas phosphorylated levels of these two proteins were missing in unstimulated cells (Figure 2a).

To further characterize the role of the PI3K/Akt pathway in TIGAR protein expression, we exposed ConA-treated lymphocytes to two different inhibitors: LY294002 (PI3K inhibitor) and Akti-1/2 (Akt inhibitor). We found that TIGAR induction in response to ConA treatment was significantly prevented in the presence of PI3K and Akt inhibitors, both at 24 h and 48 h (Figure 2b), thus indicating that the PI3K/Akt pathway was required for TIGAR protein expression in our model.

In order to assess the effect of PI3K/Akt pathway inhibitors in lymphocyte proliferation, analysis of PCNA protein levels was performed. PCNA expression was lower in ConA-treated lymphocytes in the presence of Akti-1/2 or LY294002, compared to lymphocytes treated with ConA alone (Figure 2c). Therefore, inhibition of the PI3K/Akt pathway was proven to be sufficient to block the mitotic effect of ConA in these cells.

### 2.3. TIGAR Knockdown Decreases the PCNA Proliferation Marker in ConA-Stimulated Lymphocytes

**Figure 3:**
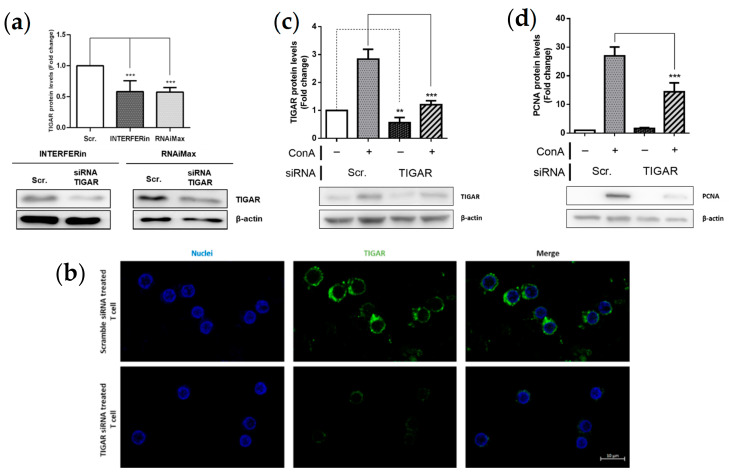
TIGAR silencing in ConA-stimulated lymphocytes. (**a**) Western Blot analysis and quantification of TIGAR silenced lymphocytes with two different reagents (INTERFERin and RNAiMax) for 48 h. Representative Western blot image is shown. (**b**) Images of immunofluorescence analysis of silenced TIGAR lymphocytes with INTERFERin reagent. (**c**) Western blot analysis and quantification of silenced TIGAR lymphocytes in response to 10 µg/mL ConA (48 h). Representative Western blot images are shown (**d**) Western blot analysis and quantification of PCNA in silenced TIGAR lymphocytes in response to 10 µg/mL ConA (48 h). Representative Western blot image is shown. Quantification of each protein level was normalized to β-actin levels. All data are presented as a mean fold change relative to untreated cells ± SEM. Differences were calculated with two-way ANOVA and Tukey’s multiple comparisons test with a normal-based 95% CI. Significant *p* values are indicated; ** *p* < 0.01; *** *p* < 0.001; *n* = 3.

To understand the role of TIGAR in stimulated lymphocytes, we depleted *TIGAR* expression in these cells. With this aim, a small interfering RNA (siRNA) protocol was optimized for transfecting lymphocytes. Two different transfection reagents, RNAiMax and INTERFERin, were used to transfect lymphocytes. TIGAR protein levels were significantly decreased by both reagents (Figure 3a). Additionally, TIGAR protein knockdown was confirmed by immunofluorescence (Figure 3b). INTERFERin was selected for subsequent experiments.

We next combined the blockade of *TIGAR* expression with ConA treatment. Lymphocytes were transfected 24 h prior to ConA treatment and TIGAR protein levels were assessed by Western blot 48 h after treatment. Under ConA stimulation, TIGAR expression was significantly lower in cells transfected with *TIGAR*-targeting siRNA compared to cells without siRNA (Figure 3c). TIGAR-depleted cells treated with ConA showed a trend towards higher TIGAR protein levels compared to untreated cells, which might be explained due to incomplete *TIGAR* silencing by siRNA.

To test the effect of TIGAR depletion on lymphocyte proliferation, total levels of PCNA were analysed in cells expressing unaltered levels of TIGAR or in TIGAR-depleted cells in the presence or absence of ConA. First, we observed that blockade of *TIGAR* expression did not affect PCNA protein levels in untreated lymphocytes, indicating that the presence of TIGAR in unstimulated cells does not impact cell proliferation. Conversely, significant reduction of PCNA expression was observed in TIGAR-depleted cells treated with ConA (Figure 3d). These results suggest that TIGAR plays a role in the expression of proliferation markers such as PCNA in human lymphocytes.

### 2.4. TIGAR Controls ROS Levels in ConA-Stimulated Lymphocytes

One of the main roles of TIGAR is to control cellular oxidative stress by reducing ROS levels [18]. The inhibition of intracellular ROS production is considered necessary to promote cell survival during and after oxidative stress [12,24].

**Figure 4:**
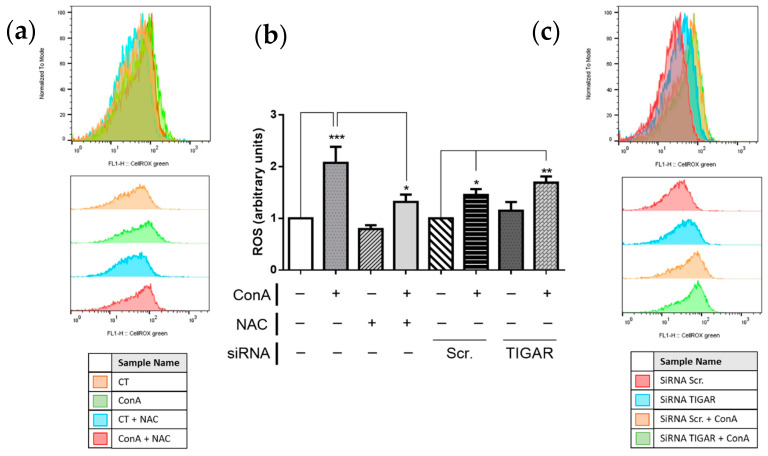
Analysis of ROS levels in silenced and antioxidant treatment lymphocytes after antioxidant treatment or *TIGAR* knockdown. (**a**,**c**) Raw data of CellROX Green fluorescence peaks of a representative experiment. (**b**) Cytometry analysis of ROS levels in T lymphocytes in different conditions; 20 mM NAC, 10 µg/mL ConA, and 75 nM siRNA TIGAR. The results are presented as a mean of all the measures. Differences were calculated with two-way ANOVA and Tukey’s multiple comparisons test with a normal-based 95% CI and ± SEM. Significant *p* values are indicated; * *p* < 0.05; ** *p* < 0.01; *** *p* < 0.001; *n* = 3.

In our model, the depletion of TIGAR-altered intracellular ROS concentration is assessed by flow cytometry using CellROX™ Green Reagent probe. Results showed that ConA significantly increased ROS levels in T lymphocytes, which is expected under proliferative conditions. Co-treatment of ConA with the antioxidant molecule N-acetyl cysteine (NAC) prevented the formation of ROS in lymphocytes in response to ConA. Abrogation of *TIGAR* expression slightly increased ROS levels in both untreated cells and ConA treated cells compared to their corresponding controls with unaltered *TIGAR* expression (Figure 4).

### 2.5. TIGAR Knockdown Results in Decreased Activity of G6PDH

Since TIGAR is known to be a regulator of PPP [12], we wondered whether the increased ROS levels observed after *TIGAR* knockdown were explained by changes in the PPP. To this aim, we assessed G6PDH activity in lymphocytes after ConA treatment in the presence or absence of *TIGAR* expression.

**Figure 5:**
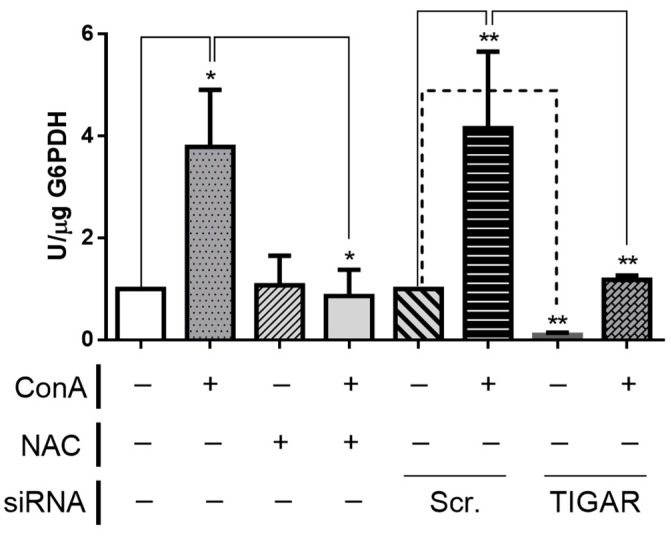
PPP activity in silenced T lymphocytes. Metabolic analysis of enzymatic activity of G6PDH in *TIGAR*-silenced T lymphocytes; NAC was used at 20 mM, ConA was used at 10 µg/mL, siRNA TIGAR was used at 75 nM. The activity is presented with U/µg and the results are normalized to protein levels. Differences were calculated with two-way ANOVA and Tukey’s multiple comparisons test with a normal-based 95% CI and ± SEM. Significant *p* values are indicated; * *p* < 0.05; ** *p* < 0.01; *n* = 3.

G6PDH activity was significantly increased in ConA-treated lymphocytes. Under NAC exposure, the activity of G6PDH was reduced to the levels of untreated cells. The abrogation of *TIGAR* expression in untreated cells led to reduced activity of G6PDH. Interestingly, TIGAR depletion significantly reduced G6PDH activity in ConA-treated lymphocytes in a similar manner as NAC (Figure 5). These results confirm the direct contribution of TIGAR to the PPP in human lymphocytes and suggest that modulation of G6PDH might not be directly linked to ROS homeostasis in these cells, since *TIGAR* blockade has an important effect on G6PDH activity (Figure 5), whereas it has mild effects on ROS levels (Figure 4).

### 2.6. TIGAR Prevents Autophagy in ConA-Stimulated Lymphocytes

**Figure 6:**
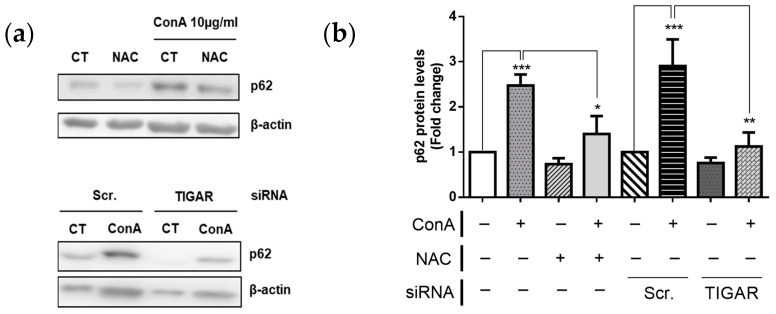
p62 protein expression in human ConA-stimulated lymphocytes after *TIGAR* depletion. (**a**) Western blot analysis of p62 protein levels normalized to β-actin in 10 µg/mL in ConA-treated and untreated (Ct) T lymphocytes ± NAC and siRNA-treated T lymphocytes. Representative Western blot images are shown. (**b**) All data are presented as the mean fold change relative to untreated cells ± SEM. Differences were calculated with two-way ANOVA and Tukey’s multiple comparisons test with a normal-based 95% CI. Significant *p* values are indicated; * *p* < 0.05; ** *p* < 0.01; *** *p* < 0.001; *n* = 3.

Linked to its role on decreasing ROS levels, TIGAR is known to regulate autophagy [26]. To characterize this, we assessed the protein levels of p62, a protein marker of autophagy inhibition, in lymphocytes expressing endogenous levels of TIGAR and in *TIGAR*-depleted lymphocytes under ConA treatment. Levels of p62 were significantly increased in ConA-treated lymphocytes (Figure 6), indicating an inhibition of the autophagic cascade in parallel to the increase of TIGAR. Both NAC and depletion of *TIGAR* expression, which both reduce G6PDH activity, led to a significant reduction of p62 protein levels (Figure 6), indicating a rescue of the autophagic pathway. These results support TIGAR role in lymphocytes autophagy inhibition.

## 3. Discussion

During T lymphocyte activation and proliferation, a rapid and programmed upregulation of metabolism is required to meet the increasing demands of energy and biomolecules needed for growth and clonal expansion. Among the metabolic changes undergoing in T cells, increased uptake of glucose and oxidation through glycolysis is central to increase ATP production [3,5]. Increased expression of Glut-1, HK-2, or PFK-2/FBPase-2, regulators of rate-limiting steps in glycolysis, is observed in activated T cells [6,10]. Together with enhanced glucose metabolism, the activity of enzymes involved in glutaminolysis and mitochondrial metabolism is also increased in order to fuel the TCA with intermediates for anabolism. In this context, a previous work from our group described the contribution of PFKFB3, the gene encoding for the PFK-2/FBPase-2 isoform with the largest kinase/phosphatase activity ratio, in the activation and proliferation of human T cells in response to TCR stimulation by ConA, phytohemagglutinin (PHA), and lipopolysaccharide (LPS) [10]. In the present work, we used stimulation by ConA to explore the role of TIGAR, a key enzyme regulating glycolysis and the PPP [12], in human T lymphocytes. Here we show that the expression of TIGAR is induced in cells after mitotic stimulation. The increase in TIGAR levels was found to be PI3K/Akt pathway dependent, since PIK3 and Akt blockage prevented TIGAR upregulation. These findings are concordant with those previously described, where we found that PFKFB3 upregulation in T lymphocytes activation was controlled by the PI3K/Akt signalling pathway [10]. Overexpression of both PFKFB3 and TIGAR has been observed in several tumours [8], where parallel increases of glycolysis and the PPP provide cells with enhanced ATP, Ribose-5-P, and TCA intermediates required for biosynthesis, together with NADPH to control ROS levels.

Despite the role of TIGAR in cancer cell metabolism being widely explored since 2006 [12], very little is known about the regulation of this enzyme in healthy tissues under physiological conditions. However, the high similarity between the metabolic transformation that occurs during oncogenesis and lymphocyte proliferation can explain why some glycolytic enzymes, such as PFKFB3 and TIGAR, are upregulated both in malignant cells as well as in activated T lymphocytes. T cells shift from an OXPHOS-based metabolism in their quiescent state towards a highly glycolytic metabolism, a scenario in which TCA intermediates are mainly used for anabolism, whereas ATP is mostly generated in glycolysis [3]. Our results indicate that in this highly glycolytic context, TIGAR upregulation allows for the redirection of part of the G6P to the PPP. Several molecules are capable of inducing T cell activation in vitro though TCR stimulation, from among which we used ConA. In a previous publication, our group showed that ConA increases the expression of CD25 in the membrane of T cells and triggers cell proliferation, evidenced by a reduction in CFSE staining, while the expression of PFKFB3 and PCNA were increased [10]. Under similar conditions, we show here that TIGAR expression is increased in a PI3K/Akt-dependent manner. This is in accordance with previous results from our group which showed that in the cervical carcinoma cell line, HeLa inhibition of Akt prevented the induction of TIGAR [24]. The axis PI3K/Akt/mTOR is one of the most important pathways driving T cell activation and regulating anabolic metabolic reprogramming in T cells exiting quiescence [23] and it was previously shown to be necessary for the upregulation of PFKFB3 after ConA treatment [10]. The results presented here indicate that this axis is required for the upregulation of TIGAR and PCNA. To further study the role of TIGAR in stimulated lymphocytes, *TIGAR* expression was disrupted by siRNA. Transfection of primary lymphocytes is methodologically challenging and the percentages of effective inhibition of mRNA expression are variable between samples. Overall, we reached a 50% depletion of *TIGAR* expression. Interestingly, lymphocytes in which *TIGAR* expression was impaired showed decreased levels of the proliferation marker PCNA, indicating that the induction of TIGAR that follows TCR stimulation contributes to lymphocyte proliferation. This is consistent with previous studies that showed the pro-survival role of TIGAR in haematological malignancies. Depletion of *TIGAR* expression by siRNA decreases the percentage of senescent cells in adult T-cell leukemia cells in response to low doses of Nutlin-3a [27]. As inhibition of apoptosis is one of the key features of senescent cells, depletion of TIGAR expression may result in increased apoptosis in these cells due to impaired senescence. In an in vivo xenograft model of human T-cell leukemia virus type-1 (HTLV-1)-induced T cell lymphoma, TIGAR was highly expressed. Additionally, its expression was found to counteract oxidative stress, mitochondrial damage, and cytotoxicity caused by viral proteins. *TIGAR* depletion by siRNA in T lymphocytes infected with HTLV-1 produced an increase in oxidative stress and the overexpression of FLAG-tagged TIGAR inhibited ROS production of these cells [28]. In solid tumours, this association between TIGAR expression and ROS production has been widely described and it has been attributed to the role of TIGAR in potentiating the PPP [8,12]. Some of these studies have pointed out the involvement of TIGAR in different stages of disease progression, such as colorectal cancer [29]. It has been recently described that TIGAR is required for pancreatic cancer cells in tumour initiation and development of established metastases [30]. Other studies have also pointed out the possibility of using TIGAR as a therapeutic target for cancer treatment. In glioma cells, downregulation of *TIGAR* expression by siRNA results in radiosensitization [31,32]. In breast cancer, TIGAR overexpression allows malignant cells to increase mitochondrial metabolism by using extracellular lactate to fuel TCA [33], providing evidence for a role of TIGAR beyond glycolysis redirection and the PPP.

In this study we provide evidence that the activities of TIGAR and G6PDH, the first enzyme of the PPP, are linked in healthy lymphocytes. An increase in G6PDH was observed in ConA-treated lymphocytes, which was completely prevented by the disruption of TIGAR expression. This led to a decrease in the production of Ribose-5-P, which can explain the decreased proliferation of ConA-treated TIGAR-depleted cells. However, ROS levels were increased in ConA-treated cells regardless of a high activity of G6PDH, which might reflect a compensatory response. This was additionally supported by observing that NAC treatment, which restores GSH availability, decreased ROS levels and, consequently, G6PDH activity. TIGAR-depleted cells did not show an increase in ROS levels comparable to the observed downregulation of G6PDH activity, suggesting that other antioxidant mechanisms besides the PPP control ROS homeostasis in human lymphocytes. ROS have been described to boost T lymphocyte activation by coupling the increased mitochondrial activity, which produces H_2_O_2_, to interleukin 2 (IL-2) expression [34]. Finally, the increase of ROS in ConA-treated lymphocytes was found to be accompanied by decreased autophagy, which was rescued by TIGAR depletion. The observation that TIGAR inhibition and NAC have similar effects regarding autophagy modulation while NAC decreases ROS and TIGAR inhibition slightly increases ROS in ConA-treated cells might indicate that the role of TIGAR in autophagy control is not dependent on its ability to modulate ROS levels. The link between TIGAR and autophagy has been discussed and can differ according to the cellular context. Our results indicate that in lymphocytes TIGAR can prevent autophagy, as it was previously shown in cancer cells [19,22,23].

The work we present here situates TIGAR in the context of the metabolic rewiring of lymphocytes, and more specifically as a key player in the redirection of G6P to the PPP pathway to produce Ribose-5-P and NADPH. Analogously to what occurs in cancer cells, the expression of *TIGAR* and *PFKFB3* allows for a high glycolytic flux together with increased capacity for DNA synthesis and control ROS levels in activated lymphocytes. In the context of infection, increased expression of TIGAR and the PPP in lymphocytes can contribute to increased capacity of these cells to maintain ROS homeostasis, allowing for the roles as secondary messengers of these radical species while at the same time preventing ROS-induced cell death. Consequently, TIGAR expression contributes to decrease stress levels in lymphocytes that undergo clonal expansion, contributing to the acquisition of their effector functions and proper function of the cellular immunity.

## 4. Materials and Methods

### 4.1. Lymphocyte Sample Collection

Culture lymphocytes techniques were set up in Simon-Molas et al. (2018). Lymphocytes were provided by the group of Dr. Nuria Lloberas as leucoplatelet blooded concentrates of healthy blood donors from Hospital del Mar, under protocols approved by the Ethics Committee of Bellvitge University Hospital and with the 1964 Helsinki Declaration.

### 4.2. Lymphocyte Purification

Human peripheral blood mononuclear cells (PBMCs) were isolated using a Ficoll density gradient. It was performed using Ficoll-Paque Plus from Healthcare which was added to the blood in 1:2 dilutions. Around 1 × 10^8^ cells/mL were resuspended and rested during 2 or 3 h in X-VIVOTM medium from Lonza and 2% of human serum (Sigma Aldrich, Taufkirchen, Germany) (37 °C, 5%, CO_2_, 70–80% humidity) in Corning^®^ cell culture 175 cm^2^ flasks from Sarstedt. The isolation of lymphocytes was performed using a double-sided adhesion protocol. Non-adherent cells or lymphocytes were collected and cultured with completed RPMI 1640 medium (Biological Industries, Kibbutz Beit-Haemek, Israel) from 10% of inactivated foetal bovine serum (FBS), 1% of penicillin-streptomycin, and 1% of L-glutamine at 2 million of cells/mL (37 °C, 5% CO_2_, 70–80% humidity) in 75 cm^2^ flasks from Sarstedt (Nümbrecht, Germany).

### 4.3. Lymphocyte Treatments and Sample Collection

Lymphocytes were activated using ConA (10 µg/mL). Concanavalin A is a plant mitogenic lectin that binds directly to cell membrane polysaccharides and activates T lymphocytes by binding to their receptors [35]. Thirty minutes before the addition of ConA, stimuli like Akti-1/2, Ly294002, N-Acetyl-L-cysteine (NAC), at doses of 10, 20, and 20 µM respectively, were added. Reagents and stimuli are from Sigma Aldrich, Taufkirchen, Germany. Samples were collected at 24 h and 48 h for cells treated with ConA. Four milliliters of the cell culture were placed in sterile tubes from TPP^®^ and centrifuged at 1200× *g* rpm for 5 min.

### 4.4. Lymphocyte Transfection with siRNA

Small interfering RNAs (siRNAs) were designed according to criteria outlined elsewhere. Specificity was checked by BLAST. Transfections were carried out using three Stealth siRNAs (Invitrogen Corp. Thermofisher Scientific, Waltham, MA, USA) sequences targeted against *TIGAR* (‘‘TIGAR-siRNA’’) (T1: 50-GAAGUUAAACCAACGGUUCAGUGUA-30, T2: 50-CAGGAUCAUCUAAAUGGACUGACUG-30, and T3: 50-CAAGCAGCAGCUGCUGGUAUAUUUC-30) and two medium GC negative control Stealth siRNAs (‘‘Scrambled siRNA (Scr.)’’) (50-GAAGUUAAACCAACGGUUCAGUGUA-30 and 50-CAGGAUCAUCUAAAUGGACUGACUG-30). Cells were plated at a density of 2.5 × 10^5^ cells in 6-well plates and allowed to attach overnight. Cells were then transfected using different reagents diluted in Opti-MEM Reduced-Serum Medium (GIBCO, Thermofisher Scientific, Waltham, MA, USA). The final siRNA concentration was 75 nM. After 4 h, complete media was added to each well. Twenty-four hours after transfection, cells were trypsinized, resuspended in fresh media, and re-plated for clonogenic cell survival and cell viability assays.

T lymphocytes were transfected with 5 different transfection reagents to check which gave the best results of silencing *TIGAR* expression. The reagents used were: Lipofectamine 3000 Reagent, RNAiMax Transfection, Oligofectamine Reagent, Polyplus Transfection, and INTERFERin siRNA Transfection Reagent (Thermofisher Scientific, Waltham, MA, USA).

Tubes were of 2–3·10^6^ lymphocytes per mL. An amount of 75 nM *TIGAR* siRNA per plate was added. The required doses for each reagent are given in the protocol of each reagent. The transfection was left for a total of 72 h. Over time, lymphocytes were collected and deposited in sterile TPP^®^ tubes, and centrifuged for 5 min at 1200× *g* rpm.

### 4.5. Inhibition of PI3K-Akt Signaling Pathway

Akti-1/2 and LY294002 (Catalog Numbers #124018 and #440202, respectively, Calbiochem, San Diego, CA, USA) were added to the cultures of T lymphocytes 30 min before ConA treatment at doses of 10 and 20 µM, respectively.

### 4.6. Protein Extraction and Analysis

Four milliliters of lymphocyte culture (approximately 8 million cells) were collected and 100 μL of Western blot lysis buffer TRIS-HCl were added. A BCA analysis was performed to determine the protein concentration of the samples. The BCA Protein Assay Kit from Thermofisher Scientific, Waltham, MA, USA was prepared using the manufacturer’s instructions. Equal amounts of protein (30–40 μg) were analysed in 12% SDS-polyacrylamide gels. Membranes were incubated with primary antibody diluted in BSA-AzNa (1:200–1:1000) overnight at 4 °C. The membranes were incubated for 1 h with the secondary antibody and images were obtained using Fujifilm LAS 3000 Intelligent Dark Box IV. The densitometric analysis was performed using MultiGauge software (Multi Gauge software V3.0, FujiFilm, Fuji photo film Co., Ltd., Tokyo, Japan) and results were normalized using β-actin protein levels.

### 4.7. Metabolic Determinations

PPP (pentose phosphate pathway) activation was assessed by the activity of the G6PDH enzyme. For the analysis, the conversion of NADP^+^ to NADPH was used. Extracellular Glucose-6-P was measured spectrophotometrically in 1 mL of cultured lymphocytes under different conditions. G6PDH was normalized to protein concentration, as determined by the Bradford assay (Bio-Rad, Hercules, CA, USA)

### 4.8. Immunohistochemistry

Lymphocytes were collected at 24 and 48 h. They were resuspended in PBS and incubated over poly-lysine-coated coverslips using poly-L-Lysine from Sigma for one hour. Cells were then fixed with paraformaldehyde 4% overnight. The next day, primary antibodies were added and kept overnight. On the third day, secondary antibodies were incorporated and nuclei were stained with DRAQ5. Images were acquired with a Spectral Confocal Microscope (TSC-SL; Leica Microsystems, Wetzlar, Germany), using a Plan-Apochromat 63X/1.4 N.A. immersion oil objective (Leica Microsystems). Excitation laser beams were 633 nm for DRAQ5 and 490 nm for Chicken anti-Rabbit (IgG) Alexa Fluor 488. Images were captured with Leica confocal software from Leica Microsystems and merged using Photoshop^®^.

### 4.9. Redox Analysis

An amount of 0.5 mL of lymphocyte culture (approximately 800.000 cells), previously treated with ConA and/or NAC, was collected and washed twice with 0.5 mL of sterile PBS and centrifuged at 1200× *g* rpm for 5 min. Five-hundred microliters of Hank’s Balanced Salt Solution (HBSS) medium from Sigma Aldrich (Taufkirchen, Germany), and CellROX™ Green Reagent (Thermo Fisher Scientific, Waltham, MA, USA) was added to the tubes at a final concentration of 1 μM. After incubation at dark for 30 min, cells were washed with sterile PBS, centrifuged for 3 min at 1200× *g* rpm and carried to flow cytometry analysis using a BD FACSCanto II Cytometer with FACSDIVA software (BD Biosciences, Bedford, MA, USA).

### 4.10. Statistics and Data Analysis

To analyse the data obtained, parametric tests for matched samples were performed. The unpaired *t*-Student test was used to establish significant differences between TIGAR’s expression in controls versus treated cells. To analyse the differences between more than two groups, a one-way ANOVA test was performed. For time courses, a two-way ANOVA test was performed. Significance level was set as a *p*-value < 0.05 and at least three different sample buffy coats were necessary to establish significant differences. Calculations were performed using GraphPad for Windows, version 6.0. It was obtained from GraphPad software from La Jolla, CA, USA.

## 5. Conclusions

**Figure 7:**
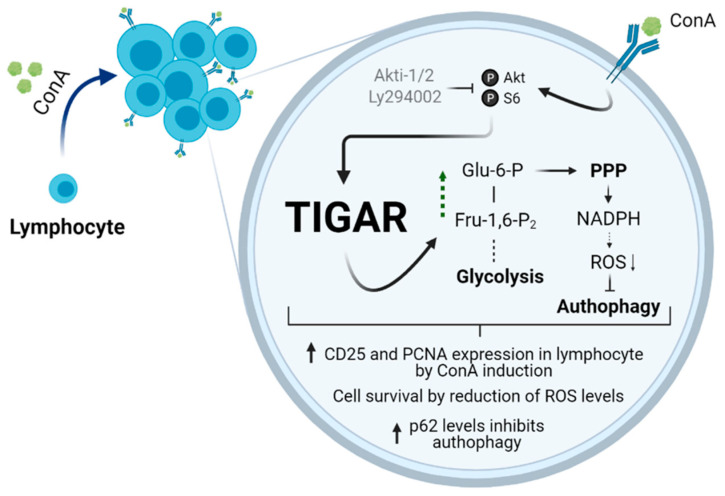
Graphical abstract of the main findings of this work. Mitotic agents such as ConA induce TIGAR in a PI3K/Akt-dependent manner. The activation of this pathway promotes cell survival, reduction of ROS by increasing PPP, and cell proliferation. Abbreviations: ConA, Concanavalin A; TIGAR, TP53 induced glycolysis and apoptosis regulator; Glu-6-P, glucose-6-phosphate; PPP, pentose phosphate pathway; Fru-1,6-P2, fructose-1,6-bisphosphate; ROS, reactive oxygen species. Created in BioRender.com (accessed on 21 May 2021).

In summary, the expression and protein levels of TIGAR are increased in human lymphocytes stimulated with ConA. This effect is mediated by the PI3K/Akt pathway. This results in increased carbon flux to the pentose phosphate pathway (PPP) and decreased ROS levels. Consequently, TIGAR offers protection from oxidative stress and prevents autophagy in lymphocytes (Figure 7).
